# Technology and responsibility: a discussion of underexamined risks and concerns in Precision Livestock Farming

**DOI:** 10.1093/af/vfz056

**Published:** 2020-01-10

**Authors:** Ian Werkheiser

**Affiliations:** Department of Philosophy, University of Texas Rio Grande Valley, Edinburg, TX

**Keywords:** animal welfare, ethics of engineering, ethics of technology, precision livestock farming, sustainability

**Implications**

- Precision Livestock Farming (PLF) promises to replicate at scale, the care usually provided by farmers who know their animals.
- This suite of current and developing technologies has the potential to address many problems facing modern farms.
- Many underexamined concerns still exist around PLF, some of which are common to many new technologies, and others of which are more specific to these technologies being implemented on farms with humans and nonhuman animals.
- Though these concerns are not a sufficient reason to abandon PLF, they ought to be considered more carefully by everyone working on developing, implementing, or legislating these technologies.

## Introduction

Precision Livestock Farming (PLF) and similar technologies are a vibrant and growing sector of technological development, and increasing importance to those involved in livestock production and food systems. Though they hold significant promise, they also contain some serious dangers as well as unavoidable costs. In what follows, I will discuss some of the work me and others have been doing on the topic of these risks, in the hopes that they can be better understood by policy makers, engineers, farmers, academic analysts, and anyone with a vested interest in our interrelated food system (Piso et al., 2014, 2016; Werkheiser and Piso, 2015; Werkheiser, 2017, 2018).

**Figure 1.**
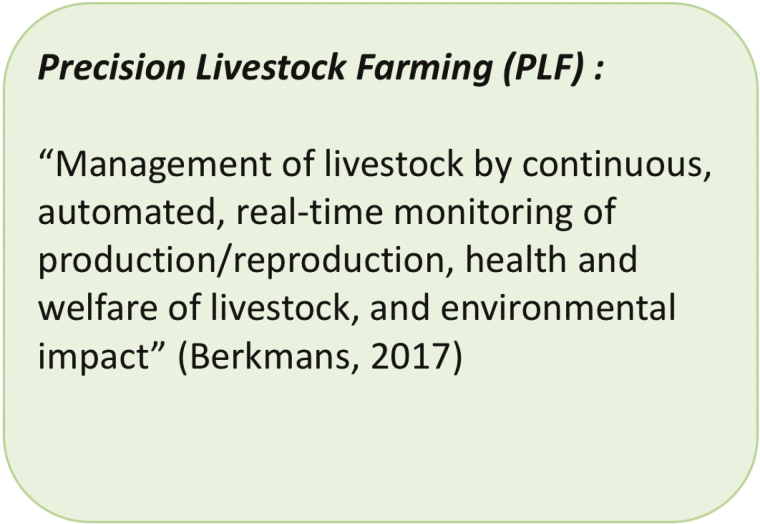
A summary of the general principles of PLF were reviewed by Berckmans (2017).

Precision Livestock Farming (often referred to as PLF) is not a single technology but rather refers to a suite of various technologies, many of which at this stage are still only speculative (Figure 1). They have the shared goal of detecting detailed and subtle information about each individual animal on a farm and using this information in management decisions. Another common term for this approach is Integrated Management Systems (often referred to as IMS), and other terms such as “smart farming” have been suggested but have not yet received wide acceptance. For consistency, I will use PLF exclusively in this review.

## Precision Livestock Farming

Some scholars have thought about PLF as an application of process engineering to the practice of livestock rearing, management, and processing (Frost et al., 2003). Process engineering looks at interconnected processes and tries to identify those processes which are under independent, open-loop control (i.e. the output is not measured or that measurement is not automatically fed back into the system as an input). Once these have been identified, as many processes as possible are given integrated controllers and put into closed-loop control (i.e. the output is automatically fed back into the system as a new input). This leads to massive increases in efficiency as well as accuracy in responsiveness as fewer decisions go through human hands. On a farm with livestock, the most vital “systems” as well as the ones most difficult to regulate are animals themselves and those centered on animals, such as feeding. This is because animals are what process engineers describe as complex, individual, and time-variant. This means that it is necessary to continually monitor them for a wide variety of variables, predict how those variables will change in the future based on current states and possible interventions, and conduct those interventions to alter the current and future states. Doing so without modern technology requires intensive contact-hours with well-trained personnel, but PLF attempts to replicate and perhaps even improve this through automated processes and technological innovation.

Like many new technologies, when PLF is introduced to a new audience, it is often framed as a solution to pressing problems. For our purposes of understanding and critically reflecting on PLF technologies, it is worth looking at what particular problems these technologies are meant to address (Berckmans, 2014, 2017).

Livestock agriculture faces a growing problem, and it is a problem of growth. Global demand for meat and other animal products is increasing and shows no signs of slowing down. Indeed, when people move from extreme poverty to merely being poor, the main thing that they spend their “second euro per day” is on animal protein (Thompson, 2015, p. 149). This means that in a best-case scenario where global poverty is massively alleviated, even in the absence of predicted increases in the global population, the rise in demand for animal products would concomitantly increase. At the same time, as demand is increasing, market forces and economies of scale are also pushing farms to consolidate and get bigger.

Taken together, this leads to a situation where more and more animals are being managed by fewer and fewer farmers. This leads to some benefits, such as increasing efficiency, but the problems associated with this approach are potentially serious, including increased density of livestock production exacerbates the environmental impact of animals on the climate, ground water and water quality, and air quality; problems of animal welfare as animals become stressed from the close quarters of high-capacity operations as well as welfare problems arising from the decrease in attentive care necessitated by scaling up; economic sustainability problems as economic benefits are offset by livestock loss to the welfare problems leading to smaller profit margins on farms which often have had to take on a debt burden in order to grow; problems of social sustainability as the communities around farms shrink due to general trends toward urbanization as well as the particular effects of farm consolidation removing potential reasons to stay and the financial means to do so; and a loss of identity—as the management of farms becomes more of a technocratic exercise, the romantic image of livestock farmer as careful, and attentive steward is increasingly unrealistic, in ways which are a likely unwelcome change to many farmers and which may well affect public perception of farming with possible policy implications.

The central problem here is one of scaling up farms while still retaining individualized care and attention to particular animals, and it is this problem to which PLF is introduced as the solution. The key is the promise in PLF to be able to monitor and manage “each individual” animal. A good way to think about the importance of this is in the context of pig feed. Currently, on a large-scale feeding operation, it is necessary to feed pigs approximately the same amount, despite the fact that individual feeding requirements can vary based on age, health, and individual differences. This is because monitoring those variations would be logistically impossible or prohibitively expensive to hire enough labor. The most common solution to this is to feed all the pigs at or near the requirements for the pigs who eat the most because this maximizes weight gain for the animals. This solution leads to various problems. Welfare problems arise from unhealthy pigs, both those who are overfed and the few who are underfed. Environmental problems arise from increased methane and nitrogen production in the pigs’ waste and food waste itself, as well as from the necessity of producing and shipping feed. Economic sustainability problems arise because the purchase of unnecessary feed hurts the profitability of the operation, as well as the loss of potential weight from those few pigs who do need more, and a lower quality of meat at slaughter for the consumer also leading to lowered profits for the farmer. All this might suggest that farmers ought to feed their pigs at an average amount instead of a maximal amount, but that proposal is unlikely to be adopted by farmers. This is because avoiding the problems listed above comes at the cost of reduced weight gain, something which may not make economic sense, and which even if it is profitable (by saving on feed, disposal, etc.) it would need to be by enough of a margin to overcome biases toward increasing production rather than efficiency. It is also worth mentioning that if pigs were fed an average amount, this would still be too much food for some, and not enough for others—an example of the common situation in which outliers suffer from welfare problems.

PLF promises a way out of this conundrum. Through individual monitoring of each pig, they can be fed to precisely match their needs. Going further, engineers are working on ways to monitor individual pigs in a higher resolution capacity than merely the amount each one eats. For example, biosensors to detect pathogens in the air or the stool, microphones to pick up vocalizations, electrodes to detect skin conductivity and heart rate, automatic scales combined with volumeters to measure lean-fat ratios, pedometers to predict estrus, cameras to detect position in stalls, and olfactory receptors to detect illness could all generate data that could be used to modify the amount of feed, timing, and additives in ways to benefit an animal’s welfare as well as maximize profits for the livestock producer. Similar PLF improvements could be made in other areas of animal management, including light levels, temperature, medicine, breeding, and more (Vranken and Berckmans, 2017).

**Figure 2.**
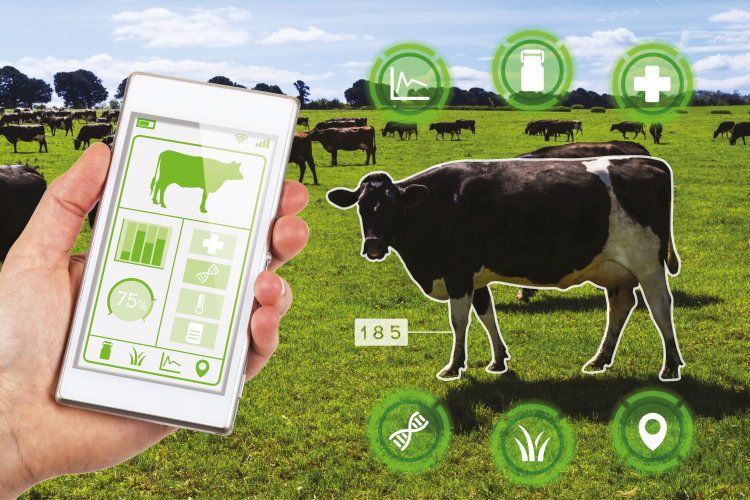
Potential use of apps for smart phones or other hand-held devices to monitor the health and well-being of individual animals on the farm.

In addition to practical benefits, PLF also promises to ameliorate the loss of identity of farmer as conscientious stewards. One way in which this can be done is by having the information generated by the sensors available to farmers or farmworkers via mobile devices. Observations, judgments, and adjustments could be made by humans, either in person or by using monitoring technology. Indeed, advocates of PLF often stress the possibility of access by farmers, for example, by promoting an app for their phone that allows them to monitor individual animals or stalls in addition to views of their entire farm (Figure 2). The downside to this would be that much of the efficiency of integrated systems and process engineering would be lost if people had to make moment-by-moment decisions, but it may still be worth it for cultural reasons. Even if decisions were automated, it is still possible for farmers to have access to the data and be much more aware of the specific needs of their animals and general trends than they are currently.

Another way some of these technologies enable people to preserve traditional identities on a modern farm (depending on how one evaluates what stewardship requires) is by making the decision to have that conscientiousness handled by the algorithms. In this way of thinking, the animals still receive all the welfare benefits of careful attention, and this is because of the will of the farmer; thus, the farmer is still discharging their duties in a meaningful way. Indeed, it is possible that decision algorithms and precise measurements could do a better job of being an attentive farmer than a human on a small farm. For example, adjustments could be made more rapidly and to a finer degree than people are capable of doing, and decision algorithms can learn over time to make predictions between variables that humans might not ever see because the causal chain is obscure. As one paper in favor of PLF says, “We can not only replace the farmer’s ‘eyes and ears’ to each individual animal as in the past, but several other variables (infections, physiological variables, stress, etc.) will soon be measurable in practice” (Berckmans, 2006), another points out, “Traditionally, livestock management decisions have been based almost entirely on the judgement and experience of the stockperson who has to estimate or guess the likely effects of any control action, taking into account the complexities of the processes involved. This leads to dilemmas” (Frost et al., 2003). A special report by the UK Farm Animal Welfare Committee states, “Precision farming, such as telemetry boluses to measure rumen pH, can detect nutritional acidosis at a subclinical level not apparent to the stockman” (Farm Animal Welfare Committee, 2016).

Some of these promised benefits of PLF are already real, and many that are currently only potential have enough promise to warrant further pursuit. However, the risks and costs of PLF remain underexamined. I have been focusing on these, and in the rest of this article, I will highlight a few of the most salient. Some of the worries are similar to those that are shared by many new technologies, while others are specific to PLF.

## Underexamined Risks

One worry common to many new technologies is that automation will lead to the loss of jobs on farms, and the deskilling of many of the remaining jobs. One of the concerns discussed above as part of the problems these new technologies are meant to solve is the lack of sustainability of farms in the modern era. Yet since the first discussions of sustainability, it has been acknowledged that part of sustainability is the social component (Thompson, 1992). If farms are run by increasingly few technical managers and deskilled laborers using automatic monitoring, there are naturally fewer ways to get into farming as a career if one does not already own a farm. This makes farming and farm communities less sustainable, and is also a harm to those farmworkers.

It is true that the jobs which remain may well be less physically demanding and safer, as well as the addition of at least a few technical jobs to operate PLF equipment. However, farm labor and jobs with a connection to food production are often seen as particularly important (Thompson, 2010, 2017), and their loss might be much more severe than in other industries. Job loss and job simplification from automation is a complex issue, and one with a wealth of often contentious literature on how it should be viewed and how (if at all) it should be ameliorated. They can be seen as an inevitable tragedy, or an avoidable harm, or a neutral reconfiguration of the economy, or an on-balance good, depending on one’s views on economics, technological development, and so on. Depending on one’s views, various solutions suggest themselves, including recompensating workers, directing technological research and development to make the problem less severe, or as an argument against implementing the technologies altogether.

Another risk which PLF shares with other technologies is the extent to which it risks consolidation, in this case, the further consolidation of farms. The concern here is that technological innovation creates a “technological treadmill” in which wealthier participants in a sector are able to benefit the most from new technologies because they can afford to adopt them and because the technologies are often designed with their needs in mind, either intentionally or due to unintentional biases of the researchers (McCune, 1998; Roling, 2009; Piso et al., 2014). This is a common problem in industrial agriculture and other industries as well. There are ways to mitigate the concern, most prominently the use of tax subsidies for smaller farms to make improvements. This has some of its own ethical and practical issues, but it seems like a potential direction at least worth exploring. Either way, these problems of economic and social unsustainability remain a serious concern to be thought through before uncritically promoting technological development.

A third concern that PLF shares with other technological advancements is one of value hierarchies, and the ways in which these are embedded in particular technologies. When proponents of these technologies discuss the many things it can help with, such as profitability for farmers, environmental issues, animal welfare, and so on, it may well be the case that these technologies can help with any of these and even marginally improve all of them simultaneously from their current states. But in extremes, these values may not be mutually maximizable. Even different conceptions of sustainability held by farmers are not necessarily mutually maximizable (Piso et al., 2016). At a certain point, there will be tradeoffs and decisions to be made about how to prioritize values. How these decisions are made, and who has input into the decision, will dictate who benefits from these technologies and who is disadvantaged by the technologies. This is not a problem for all versions of PLF (since many versions will presumably make small enough changes that the tradeoffs do not yet have much bite), but it is something that must be kept in mind as the technologies develop (Wathes et al., 2008; Lehr, 2014).

A final risk which is shared with many other technologies is the possibility of crossover between some aspects of PLF to human applications. The concern here is that research into monitoring nonhuman animals closely and training up predictive algorithms to interpret that data can have two effects relevant to humans. The first is that money is being directed to develop technology which could have a dual-purpose use to monitor humans. The second is that it normalizes the technology and thus reduces resistance to it, either in law or in public opinion. Examples of this in other areas might include commercial drones, the increasing prevalence of facial recognition technology, or monitoring built into various home electronics such as mobile devices and digital personal assistants. In the area of PLF, the most salient recent example has been discussions in China of using facial recognition technology and voice recognition technology to help with livestock management and disease prevention (Wee and Chen, 2019). There are concerns that the Chinese government might be using this as a way to roll out a technology and research it before using it in other applications.

As mentioned above, some concerns are more particular to PLF. One such is that the improvements promised by these technologies provide a cover for the consumption of animal-derived products, as large-scale industrial livestock production is once again given the romantic veneer of close attention to animal welfare, environmental impact, and so on. PLF, after all, includes monitoring animals as they go through slaughter, processing, and packaging in addition to animals alive on farms producing milk or used for services on the farm. Whether improvements to animal welfare before slaughter or during exploitation (which might further encourage animal consumption) is an unalloyed ethical problem, or an unalloyed benefit, or a mixed tradeoff, of course depends on one’s views on the consumption of animal-derived products, as well as one’s opinion on the strategies of abolition or amelioration of moral problems (Thompson, 2015).

Another issue specific to PLF has to do with animal welfare, and specifically the different, often competing, models of animal welfare. As Thompson lays out (2015, pp. 137–152), people talking about animal welfare may be referring to a host of different things, including the physical well-being of the animal, its psychological well-being, or its ability to engage in species-specific activities. To this, I think it is worth adding another model of welfare built off the good of autonomy.

All these can come apart, and it is not immediately clear which ones are best supported by PLF. As discussed above in relation to feeding, these technologies are quite likely to increase physical and medical welfare. Psychological welfare is more of an open question; as mentioned, the loss of interaction with humans might be a mixed blessing for animals, and whether PLF will increase or decrease stress is also an open question (will it allow for more or less interaction with conspecifics? Will it be unobtrusive? Will it require the animals to act differently in ways they do not like in order to be monitored?). Species-specific behavior as a means of welfare is also a partially open question. The European Agricultural Machinery Organization advocates for PLF on their website. There, they say that among other benefits (including increased productivity, but also the ability for farmers to receive updates about their herd via SMS), PLF’s “Automated solutions operate without the limitations and constraints of human labour and thus provide more freedom for animals for self-determined, species-appropriate behavior” (CEMA-AGRI.org, n.d.). There are reasons to think that it may allow animals to engage in species-appropriate behavior if these technologies are unobtrusive and allow the animals to live in ways they evolved (though again such behavior may cut against the other models of welfare), but it is also possible that these technologies will work best without species-specific behavior, for example, in a room with monitoring equipment.

The final conception of welfare just mentioned but not usually included in matrices of animal welfare approaches is autonomy, the idea that it is a welfare goal for animals to be able to make choices and realize their preferences. It is clear that this will cut against some other conceptions of welfare (many animals, when given the choice, do things which are not maximally healthy nor evolved species behaviors), but it is plausibly a good wish to maximize for anything with preferences. It is possible that PLF would harm animals along this axis—after all, automated changes to the temperature of a stall or the additives to feed seem a long way from autonomous choice (though if the animal comes to learn how to interact with the monitors to get what it wants, then these technologies would be an increase in autonomy by aiding the animal in communicating their desires).

**Figure 3.**
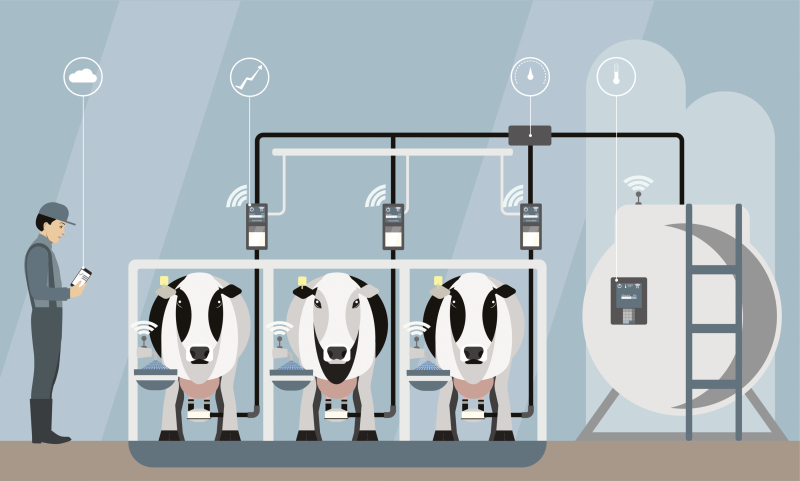
Schematic of a robotic milking facility where dairy cows can choose the time and frequency of milking.

An example of the ways in which these technologies might increase autonomy can be found in Stuart et al.’s (2013) example of robotic milking facilities, where cows can choose to be milked whenever they wish, and are rewarded with feed (Figure 3). The authors suggest that this technology allows the cows to have more autonomy and participation in the decisions affecting their lives. This technology is not usually thought of as an example of PLF, but it is similar as it replaces the need for human attention and judgment. It also differs from PLF as it does not attempt to recreate that attention and judgment. However, it does illustrate the possibility that technological developments could be an improvement to animals’ autonomous choices about their lives over modern, industrial farming approaches that treat animals as an average group member with average needs, and perhaps even over small-scale, traditional farming.

The last concern specific to these kinds of technologies is the extent to which it is possible for farmers to discharge their duties to care for their animals via these kinds of closed-loop monitoring technologies. Earlier the possibility that PLF holds out for farmers to be able to scale up close, personal attention, and individualized care for their animals to much larger operations was discussed. If we grant that farmers have that professional duty of care for the animals dependent on them, and perhaps that this is recognized by farmers and the public, we do not generally think that discharging responsibilities can be accomplished by having technological stand-ins for our own attention (consider other cases in which we have a duty to care for dependent beings, such as companion animals or even elderly people or very young human children). There are many reasons for this, and some of them are relevant to that particular dependent relationship of farmer and livestock. For example, it is quite possible that any monitoring technology will miss out on important signs that the engineers did not know farmers look for, but which can only be developed over time by building a relationship with the actual animal. This is compatible with the earlier claim that PLF technologies might also find things farmers would miss. Consider the different information available via the monitoring technology of standardized tests vs. a relationship between a teacher and a student.

**Figure 4.**
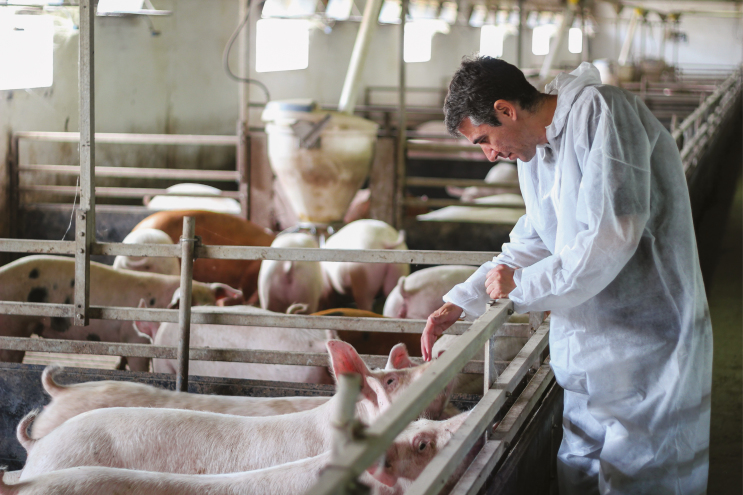
Photo of an animal care taker interacting with growing pigs.

Another reason we generally see it as insufficient is that technological fixes do not replicate an actual relationship where the dependent being known is being cared for (Figure 4). Food being distributed in a feeder is different than the food being found by the animal or given to it by a human companion. It feels to some of us that using technology in place of building an actual relationship of care is a dereliction of a duty to build that very relationship. As we have discussed at multiple points, it is likely impossible to discharge these duties on a large-scale feeding operation. But if it is true that these duties exist, telling ourselves that PLF technologies do the job just as well or better, and that we can still have the romantic idea of a caretaking farmer is perhaps false. In that case, they would be more of a poor substitute in a bad situation than they are a fix to the problems in the first place.

## Conclusion

We have seen that there is a lot of pressure from various quarters for traditional farms to scale up, yet doing so brings with it a host of problems around animal husbandry. We have also seen that PLF is a promising solution, but one with a host of concerns that are currently underexamined and underdiscussed, particularly outside academia. None of these concerns are so damning that PLF should not be pursued. However, they all require careful negotiation and forethought, and incorporation of the perspectives of many stakeholders, including farmers, farmworkers, and stockpersons, the wider communities in which farms are embedded, scientists, policy makers, and the perspectives of the animals themselves. Integrating these voices into discussions around food systems is a difficult process, but one important step is for all the participants to agree that there are concerns to discuss. It is that ground-clearing argument that I have been and am continuing to make in this article.

## About the Author

**Ian Werkheiser** is Associate Professor in the Department of Philosophy at the University of Texas Rio Grande Valley and the Director of the Center for Collaboration and Ethics. He works on issues in Environmental Philosophy, Philosophy of Food, Philosophy of Animals, and Social Epistemology. He is particularly interested in the ways expert and nonexpert communities do and could interact with each other over difficult questions, particularly around the environment, food, and nonhuman animals. He received his PhD in 2015 from Michigan State University, where he worked on questions around environmental justice.
